# MEK and the inhibitors: from bench to bedside

**DOI:** 10.1186/1756-8722-6-27

**Published:** 2013-04-12

**Authors:** Akintunde Akinleye, Muhammad Furqan, Nikhil Mukhi, Pavan Ravella, Delong Liu

**Affiliations:** 1 Department of Medicine, Westchester Medical Center and New York Medical College, Valhalla, NY, 10595, USA; 2 Division of Hematology and Oncology, New York Medical College and Westchester Medical Center, Valhalla, NY, USA

## Abstract

Four distinct MAP kinase signaling pathways involving 7 MEK enzymes have been identified. MEK1 and MEK2 are the prototype members of MEK family proteins. Several MEK inhibitors are in clinical trials. Trametinib is being evaluated by FDA for the treatment of metastatic melanoma with BRAF V600 mutation. Selumetinib has been studied in combination with docetaxel in phase II randomized trial in previously treated patients with advanced lung cancer. Selumetinib group had better response rate and progression-free survival. This review also summarized new MEK inhibitors in clinical development, including pimasertib, refametinib, PD-0325901, TAK733, MEK162 (ARRY 438162), RO5126766, WX-554, RO4987655 (CH4987655), GDC-0973 (XL518), and AZD8330.

## Introduction

The mitogen-activated protein kinase (MAPK) signaling pathways involve a family of protein kinases that play critical roles in regulation of diverse cellular activities, including cell proliferation, survival, differentiation, motility, and angiogenesis. The MAPK pathways transduce signals from various extracellular stimuli (growth factors, hormones, cytokines and environmental stresses), leading to distinct intracellular responses via a series of phosphorylation events and protein-protein interactions [1].

Four distinct MAPK cascades have been identified and named according to their MAPK module. These are extracellular signal-regulated kinase (ERK1/2), c-Jun N-terminal kinase (JNK), p38 and ERK5. Each of these cascades comprised of three sequentially acting kinases, activating one after the other (MAPKKK/MAP3K, MAPKK/MAP2K, and MAPK). These signaling cascades are often dysregulated in human cancer cells. Many small molecule inhibitors targeting various component of these cascades are moving quickly from bench to bedside [2-4]. For instance, vemurafenib is the first B-RAF inhibitor that received FDA approval in 2011 for the treatment of BRAF V600E/K mutation positive metastatic melanoma [5,6].

This review focuses on MAP2K or MAPKK component of each of the four MAPK cascades with their characteristics and the small molecule inhibitors targeting these proteins/enzymes. Mitogen-activated protein kinase or MAP2K or MAPKK are commonly known as MEK proteins.

## MEK proteins

**Figure 1:**
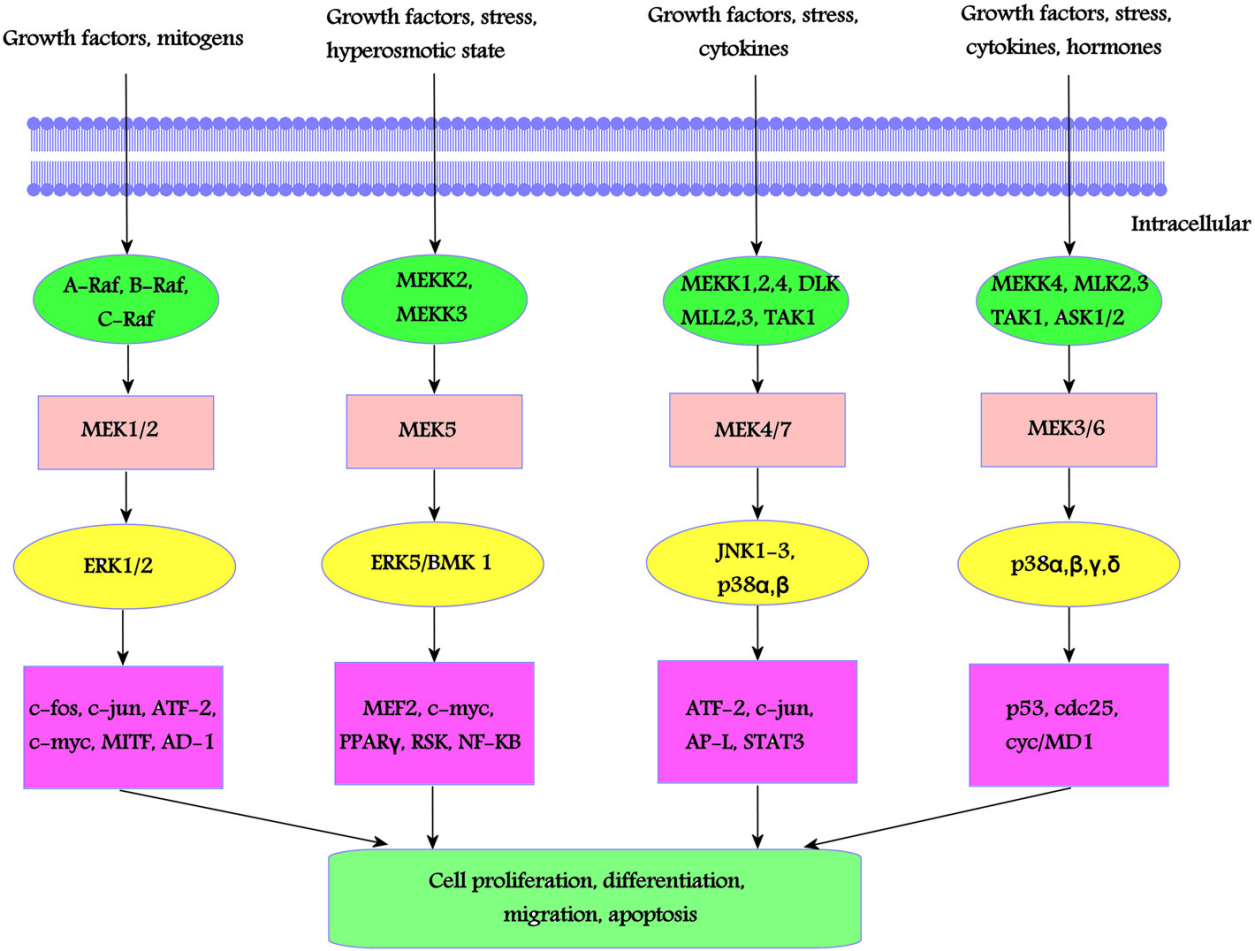
**MEK proteins and their signaling pathways.** In human, four distinct MAP kinase signaling pathways involving 7 MEK enzymes have been identified. The corresponding MEK enyzmes and their associated signaling pathways are shown in the diagram.

MEK proteins belong to a family of enzymes that lie upstream to their specific MAPK targets in each of the four MAP kinase signaling pathways and so far 7 MEK enzymes have been identified (Figure 1). These MEK enzymes selectively phosphorylate serine/threonine and tyrosine residues within the activation loop of their specific MAP kinase substrates [1].

**Figure 2:**
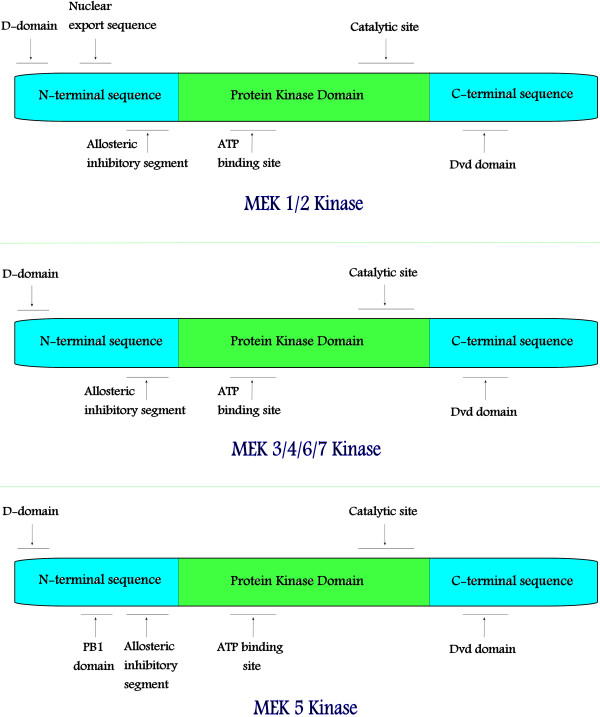
**The structures of 7 MEK proteins.** All 7 MEK proteins display a similar structural organization consisting of an amino-terminal domain, a kinase domain, and the carboxyl-terminal domain. MEKs share extensive homology in their kinase domain while the amino- and carboxy-termini are more diverse.

The molecular weight of MEK proteins ranges between 43 and 50 kDa. Like all protein kinases, they display a similar structural organization consisting of an amino-terminal domain, a catalytic domain which is also called the kinase domain, and the carboxyl-terminal domain (Figure 2). MEKs share extensive homology in their kinase domain while the amino- and carboxy-termini are more diverse.

MEK1 and MEK2 are closely related (Figure 2). They participate in the Ras/Raf/MEK/ERK signal transduction cascade. MEK 1, also designated as MAPKK-1, is the prototype member of MEK family proteins. It is encoded by the gene *MAP2K1* located on chromosome 15q22.31. The gene, *MAP2K2*, encoding MEK 2 protein, resides on chromosome 19p13.3. MEK 1/2 proteins consist of a N-terminal sequence, a protein kinase domain, and a C-terminal sequence [7]. The N-terminal sequence contains an inhibitory/allosteric segment, a nuclear export sequence (a unique feature not shared with other MAPKK family members), and a docking site (D-domain) that aids in binding ERK substrates. The kinase domain contains the ATP binding site and catalytic apparatus. The C-terminus houses the domain for versatile docking (DVD) which serves as a major determinant binding site for upstream components of the Ras/Raf/MEK/ERK cascade [8]. MEK 1/2 signaling cascade is activated by ligand binding to receptor tyrosine kinases (RTK), leading to dimerization of the receptors and autophosphorylation of specific tyrosine residues in its C-terminal region. These activated receptors recruit and phosphorylate adaptor proteins Grb2 and SOS, which then interact with membrane-bound GTPase Ras and cause its activation [9,10]. H-Ras, K-Ras, and N-Ras function as molecular switches when an inactive Ras-GDP is converted into an active Ras-GTP [11]. In its GTP-bound form, Ras recruits and activates Raf kinases (A-Raf, B-Raf, and C-Raf/RaF-1) [12]. The activated Raf kinases interact and activate MEK 1/2, which in turn catalyze the phosphorylation of threonine and tyrosine residues in the activation sequence Thr-Glu-Tyr of ERK1/2 [10]. Unlike Raf and MEK 1/2 kinases which have narrow substrate specificity, ERK1 and ERK2 have a wide variety of cytosolic and nuclear substrates. Activated ERKs can translocate into the nucleus to initiate diverse cellular responses, such as cell proliferation, survival, differentiation, motility, and angiogenesis. For instance, ERK1/2 signaling promotes the progression of cells from the G0/G1 to S phase by activation of positive cell cycle regulators cyclin D1 and c-Myc [13,14], and down-regulation of anti-proliferative proteins such as Tob1, FOXO3a and p21 [15,16]. Similarly the Raf/MEK/ERK MAP kinase pathway promotes cell survival by blocking NF-kB, leading to increased transcription of anti-apoptotic and pro-survival genes like Bcl-2 and Mcl-1 [17]. The Ras/Raf/MEK/ERK signaling is activated in human cancers via several different mechanisms. Increased ERK 1/2 signaling is often due to direct mutational activation or amplification of genes encoding key components of the Ras/Raf/MEK/ERK pathway such as Ras and B-Raf. A large-scale cancer genome sequencing study revealed that B-Raf is mutated in about 20% of all cancers and in more than 60% of melanomas [18]. Less commonly ERK 1/2 cascade can also be activated by MEKs in solid tumors including melanoma, colon, and lung carcinomas [19,20].

MEK3 and MEK6 are functionally similar and encoded by *MAP2K3 and MAP2K6* genes, respectively. The genes are both located on chromosome 17q. MEK3 and MEK6 consist of 347 and 334 amino acids residues respectively [21]. Structurally MEK6 differs from MEK3 in terms of C- and N- terminal regions. However, the ATP binding sites, and serine/threonine and tyrosine catalytic sites are conserved [22,23]. MEK3/6 signaling pathway is activated by growth factor stimulation through RTKs. Additionally, the cascade can also be activated by G-protein coupled receptors, intracellular receptors, and toll-like receptors [24], in response to numerous stimuli including physical and chemical stresses, hormones, UV irradiation, and cytokines, such as interleukin-1 and tumor necrosis factor. These stimuli activate different MAPK kinase kinases (MAPKKKs), which include TAK1, ASK1/2, DLK, MEKK4, TAO1/2/3 and MLK2/3 [25]. Active MAPKKKs phosphorylate and activate MEK3/6, which in turn catalyzes the concomitant phosphorylation of a threonine/serine and a tyrosine residue in the p38 MAPK. MEK6 activates all the four isoforms of p38 MAP kinase (α, β, γ and δ) whereas MEK3 can only activate p38α and p38β isoforms [25]. p38 MAP kinase inhibits G1/S and G2/M cell cycle progression through down-regulation of cyclin D1 and Cdc25 expression respectively, both at the level of gene transcription and post-translation [26-28]. In addition, MEK3/6-p38 MAPK cascade promotes p53-dependent growth arrest by phosphorylating p53 at serine 33 and 46 [25]. Together, these targets of MEK3/6-p38 MAPK pathway (cyclin D1, Cdc25, and p53) cooperate to arrest the cell cycle. Thus decreased p38 activity may play an important role in carcinogenesis. For example, p38 activity has been shown to be reduced in hepatocellular carcinoma in comparison to adjacent normal tissue, with tumor size inversely related to p38 activity [29].

MEK4 and MEK7 are members of the stress-activated protein kinase (SAPK) signaling cascade. MEK4, a product of *MAP2K4* gene (chromosome 17p11.2) is composed of 399 amino acids residues, whereas MEK7 is encoded by *MAP2K7* gene that maps to chromosome 19p13.3 [21]. MEK4 and MEK7 are homologous in their kinase domains which contain 11 subdomains, but their N- and C- terminal subunits are different [30]. Upon activation by upstream kinases, MAP3Ks including MEKKs (MEKK1–4), MLK2/3, Tpl-2, DLK, TAO1/2, TAK1 and ASK1/2 catalyze the phosphorylation of threonine residues in the activation segment of either MEK4 and MEK7 or MEK4 only [8,31]. Activated MEK4/7 work synergistically and activate JNK protein kinases, including JNK1, JNK2, and JNK3. To execute their functions, JNKs activate several transcription factors, including c-Jun, ATF-2, NF-ATc1, HSF-1 and STAT3 [32,33]. MEK4/7-JNK signaling pathway acts as a key tumor suppressive pathway [34,35]. It has also been reported that MEK4/7 along with its substrate JNK may promote apoptosis by phosphorylating and inactivating anti-apoptotic proteins Bcl2, Bcl-XL and Mcl-1 [36]. The MEK-JNK signaling also play an important role during embryogenesis [37]. Transgenic mice studies have shown that MEK4 activity is required for normal hepatogenesis, B and T-cell lymphopoiesis, and erythropoiesis [38,39]. There is considerable evidence that MEK4-JNK signaling cascade is also a critical mediator of cardiac hypertrophy in response to preload and afterload changes [40]. MEK4, in addition to its principal target, JNK, also crosstalks with MEK3/6-p38MAPK pathway by activating p38α and p38β [41-43]. MEK4 has been consistently observed to be inactivated by non-sense, missense or deletion mutations in many solid tumors [44]. The expression of MEK4 was shown to be down-regulated in 75% of cases of serous ovarian cancer [45]. It has been hypothesized that loss of MEK4-p38MAPK signaling cascade may be a relevant pathway associated with tumorigenesis [46].

MEK5 has 448 amino acid residues, and shares 40% identity with other protein kinases [47,48]. The upstream kinases are MEKK2 and MEKK3. Growth factors, oxidative stress and hyperosmotic conditions lead to activation of MEK5 via dual phosphorylation of its serine 311 and threonine 315 residues [49-52]. The best characterized downstream target of MEK5 is ERK5, also known as big MAP kinase 1 (BMK1) because it is twice the size of other MAPKs. The interaction of MEK5 with MEKK2, MEKK3 or ERK5 is mediated by the PB1 domain of MEK5 [53]. Upon activation, ERK5 translocates to the nucleus to stimulate the activity of a number of transcription factors [54-57]. MEK5-ERK5 signaling enhances progression through the cell cycle [58,59]. ERK5 also plays a role in cardiovascular development and neural differentiation [60,61]. Overexpression of MEK5 has been reported in cancers of the colon [62], prostate [63], breast [64], lymphoma [65], and in malignant mesothelioma [66].

## MEK inhibitors in clinical trials

A number of MEK inhibitors have progressed into clinical trials since the first MEK inhibitor (PD098059) was described in the literature in 1995 (Table 1). Currently thirteen MEK inhibitors have been tested clinically but only trametinib (GSK1120212), a selective inhibitor of MEK 1 and 2, has emerged as the first MEK inhibitor to show favorable clinical efficacy in a phase III trial [67].

**Table 1:** MEK inhibitors in clinical trials

**MEK Inhibitors**	**Target**	**Clinical trial**	**Common toxicities**	**Tumors**	**References**
**Trametinib (GSK1120212)**	MEK1/2	III	Rash, diarrhea, retinopathy	Melanoma, colorectal cancer	[67]
**Pimasertib (AST03026)**	MEK1/2	I	Nausea, rash, visual disturbance, asthenia	Colorectal, multiple myeloma	[74]
**Selumetinib (AZD6244)**	MEK1	II	Nausea, rash, xerostomia	Melanoma, NSCLC	[76-85]
**PD-0325901**	MEK1/2	I	Rash, fatigue, blurry vision, diarrhea	Melanoma, NSCLC	[89-91]
**Refametinib (RDEA119)**	MEK1/2	II	Rash	Hepatocellular cancer, melanoma, colorectal cancer	[94,95]
**TAK733**	MEK1/2	I	Not available	Melanoma, NSCLC, colorectal, breast cancer	[96,97]
**MEK162**	MEK1/2	I/II	Rash, dermatitis, CPK elevation	N-Ras melanoma, NSCLC, pancreatic cancer	[100]
**RO5126766**	Raf/MEK1/2	I	Rash, diarrhea, CPK elevation	Melanoma	[101]
**WX-554**	MEK1/2	II	Not available	Advanced solid tumors	[102]
**RO4987655**	MEK1	I	Rash, GI disorders	Melanoma	[104]
**GDC-0973**	MEK1	I	Rash, nausea, dysguesia, elevated CK	Melanoma, Pancreatic cancer, endometrial cancer	[108]
**AZD8330**	MEK1/2	I	Mental status change, rash, nausea	Advanced solid tumors	[110]

**Figure 3:**
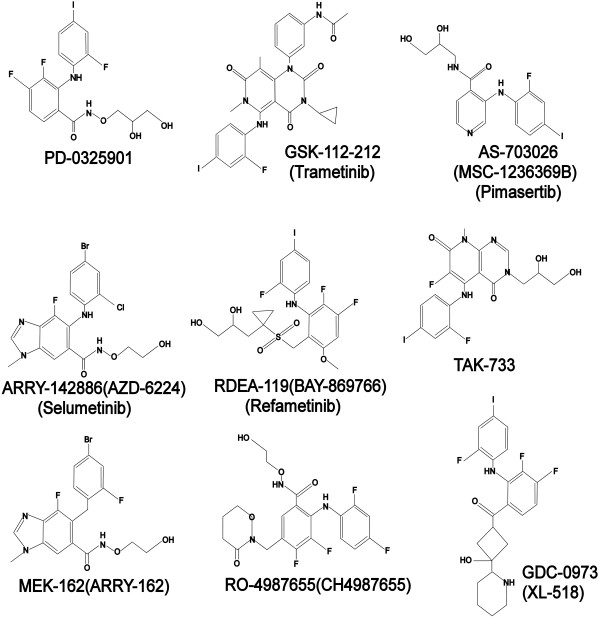
**The chemical structures of 9 MEK inhibitors in clinical development.** Nine MEK inhibitors are shown in the diagram. Among them, trametinib is being evaluated by FDA for treatment of advanced metastatic melanoma.

MEK inhibitors are sub-divided into two major classes, ATP non-competitive and ATP competitive inhibitors (Figure 3). Most of the known MEK inhibitors are noncompetitive i.e. they do not directly compete for the ATP–binding site. Rather they bind to a unique allosteric site adjacent to the ATP site. This explains the high specificity of the non-competitive MEK inhibitors [68].

## Trametinib (GSK1120212, JTP 74057)

Trametinib (GSK1120212, JTP-74057) is a potent small molecule inhibitor of MEK kinase. It is an allosteric, second generation, ATP non-competitive inhibitor with nanomolar activity against purified MEK 1 and MEK 2 kinases [69]. Preclinical studies showed efficient inhibition of p-ERK 1/2 which correlates with potent cell growth inhibition in tumor lines with mutant B-RAF or Ras. By this mechanism, trametinib induces cell-cycle arrest. In xenograft models of HT-29 and COLO205 colorectal tumor cell lines, trametinib demonstrated robust anticancer activity when administered daily for 14 days [69,70].

An early phase I dose-escalation trial of trametinib (≤3 mg/day continuous or intermittent dosing schedule) enrolled 206 patients with advanced solid tumors. Dose limiting toxicities included rash, serous central retinopathy and diarrhea. Dose of 2 mg/day was chosen for further studies. Overall objective response rate was 10%. However, B-Raf mutant melanoma had a response rate of 33% [71]. These encouraging results led to several phase II/III clinical trials of trametinib alone or in combination with other agents [NCT01553851, NCT01682083, NCT01362296, NCT01619774, NCT01245062, details are available on clinicaltrials.gov].

In the first published phase III trial of trametinib, 322 previously treated (interferon or chemotherapy) patients with advanced melanoma with V600E or V600K B-Raf mutations were randomly assigned in a 2:1 ratio to receive oral trametinib (2 mg once daily) or intravenous chemotherapy consisting of either dacarbazine (1000 mg/m^2^) or paclitaxel (175 mg/m^2^), every 3 weeks [67]. The median progression-free survival (PFS) of patients who received trametinib (4.8 months) was significantly longer than that of patients who received chemotherapy (1.5 months) (hazard ratio [HR] 0.45; P < 0.001). At 6 months, the rate of overall survival was 81% in the trametinib group versus 67% in the chemotherapy group (HR 0.54; P = 0.01).

## Pimasertib (AS703026, MSC1936369B)

Pimasertib, also known as AS703026, MSC1936369B, is a highly potent ATP noncompetitive second generation inhibitor of MEK1 and MEK2 [72,73]. Pimasertib selectively binds to the distinctive allosteric site on MEK1/2 [73]. In xenograft models, pimasertib demonstrated significant tumor growth inhibition in a human plasmacytoma H929 MM cell line at 15 and 30 mg/kg for 21 days [72]. Tumor regression was also observed at 10 mg/kg in a mouse model of D-MUT colorectal tumor [73].

A multicenter phase I/II clinical trial of pimasertib plus FOLFIRI as a second line treatment in K-Ras mutated metastatic colorectal cancer (mCRC) enrolled 16 patients [74]. Initially no DLT was observed at 45 mg/day which allowed dose escalation to 60 mg/day. At this dose, 2 of 5 patients experienced grade 3 mucositis/stomatitis leading the expansion of 45 mg/day cohort. Most common treatment-emergent adverse events (TEAEs) after 3 cycles of treatment were asthenia, diarrhea, mucositis, ocular events, nausea, rash and vomiting. These TEAEs were observed in more than one third of the treated subjects. Currently, a few phase I/II studies are underway to test pimasertib (MSC1936369B) in the setting of advanced or metastatic solid tumors including melanoma [NCT01016483, NCT01016483, NCT016968017, NCT01453387].

## Selumetinib (AZD6244, ARRY-142886)

Selumetinib is a non-ATP competitive highly selective MEK 1/2 inhibitor with IC50 of 14 nm [75]. In xenograft models, its antitumor activity correlates with decrease in phosphorylated ERK1/2 levels.

In a phase I dose escalation study of 57 patients with advanced cancers, a total daily dose of 200 mg was suggested for subsequent trials [76]. Rash, diarrhea and hypoxia were reported as major DLTs. At the recommended dose of 100 mg bid most of these TEAEs were grade 1 or 2. Other common TEAEs were nausea, fatigue, peripheral edema, transaminitis and blurry vision. Best response was stable disease and achieved in 33% of patient at the end of 2^nd^ cycle. Patients with mutated Ras or Raf remained longer in the study with higher response rate but analysis of statistical significance could not be performed due to small number of patients.

Multiple phase II studies were conducted in patients with papillary thyroid, lung, liver, pancreatic, colorectal cancers and melanoma [77-82]. Patients in these trials received selumetinib irrespective of Ras/Raf mutation status and none of these trials met their primary end points. However, patients harboring Ras/Raf mutations had higher objective response rate, indicating the need of proper patient selection in subsequent studies evaluating selumetinib.

A randomized placebo controlled phase II trial was done in previously treated patients with K-Ras mutant stage III-IV non-small cell lung cancer (NSCLC) [83]. Patients were randomized to receive docetaxel plus either placebo or selumetinib (75 mg twice daily q21 days), with overall survival (OS) being the primary end point. Median OS was 9.4 months (m) in selumetinib arm vs 5.2 m in control arm, yet the difference was statistically non-significant (HR 0.8, 80% CI 0.56-1.14, p = 0.21). However, median progression free survival was significantly prolonged in selumetinib arm (5.3 m) compared to control arm (2.1 m). Overall response rate was also better in selumetinib group. The combination of docetaxel and selumetinib had higher toxicity than docetaxel alone. Selumitinib was also studied in recurrent low grade serous carcinoma of the ovary/peritoneum in a single arm phase II study and in mitigating radioactive iodine refractoriness in metastatic thyroid cancer [84,85].

## PD-0325901

PD-0325901 is a highly specific and potent synthetic analog of MEK inhibitor CI-1040. It has subnanomolar and non-competitive inhibitory activity (IC50 = 0.33 nM) against purified MEK 1 and MEK 2 [86]. PD-0325901 inhibited phosphorylation of ERK1/2 in melanoma and papillary thyroid cancer (PTC) cell lines harboring B-Raf mutation [87]. In xenograft models, PD-0325901 demonstrated significant antitumor activity at a dose of 20–25 mg/kg/day with tumor shrinkage by 58% in PTC cells with the RET/PTC1 rearrangement [88].

In a phase I, dose-escalation study of 30 patients with multiple solid tumors, the DLTs were acneiform rash involving face, trunk and arms at 30 mg twice daily. Transient and reversible visual effects characterized by blurred vision and halos were observed at ≥15 mg BID [89,90]. The most frequent treatment-emergent adverse events (TEAE) included rash, fatigue, diarrhea, nausea, and vomiting. There were 1 PR (melanoma) and 5 SD (4 melanomas, 1 NSCLC). In an open-label, phase II study, patients with progressive, recurrent, or advanced NSCLC were treated with15 mg PD-0325901 twice daily [91]. There were no objective responses during the trial period. Due to a lack of responses coupled with the safety issues, the trial was closed after the first stage. However, Pfizer initiated a new multi-arm phase 1 study in 2012 to test PF-04691502 and PF-05212384, dual PI3K/mTOR Inhibitors in combination with PD0325901 or irinotecan in patients with advanced cancer [NCT01347866].

## Refametinib (RDEA119, BAY 869766)

Refametinib is the only cyclopropane-1-sulfonamide derivative, and exhibits a highly selective allosteric inhibition of MEK 1/2 [92]. When dosed once daily for 14 days, refametinib showed potent activity in preclinical xenografts of human melanoma A375, colon carcinoma Colo205 and HT-29, pancreatic cancer OCIP19, 21, and 23, and skin carcinoma A431 tumor models [92,93].

In a phase I/II study of patients with advanced solid tumors, refametinib was well tolerated at doses 100 mg daily. Rash was the most common TEAE [94]. Subsequently, a phase II study enrolled seventy patients to evaluate refametinib in combination with sorafenib as first-line treatment for unresectable hepatocellular carcinoma (HCC) [95]. Of sixty-five patients analyzed for efficacy per protocol, three (5%) had PR, and the median time-to-progression was 4.1 months.

## TAK733

TAK733 is a novel second-generation, allosteric kinase inhibitor with potent anti-MEK 1/2 activity [96]. In xenograft models, TAK-733 exhibited broad antitumor properties [96,97]. Phase I/II trials using TAK733 alone and in combination with alisertib in advanced non-hematologic malignancies are still accruing [NCT00948467, NCT01613261].

## MEK162 (ARRY 438162)

MEK162 (ARRY 438162) is another novel, second generation inhibitor that targets MEK 1/2 [98,99]. A phase II study examined MEK162 in 71 patients with N-Ras and B-Raf mutated advanced melanoma patients. It was given as 45 mg twice daily. Disease control rates of 63% and 51% were noticed in N-Ras and B-Raf mutant melanoma patients, respectively. No complete response was observed. Grade 3–4 adverse events include rash, diarrhea, fluid retention and creatinine phosphokinase (CPK) elevation [100]. A MEK162 analog, ARRY 300, recently completed phase I testing in healthy volunteers in the United States (NCT00828165).

## RO5126766

As a novel, highly potent, first-in-class dual MEK/Raf inhibitor, RO5126766 selectively binds to MEK 1/2 to form a stable Raf-MEK-RO5126766 complex. Cell cycle arrest was shown to be the primary mechanism for the growth-inhibitory properties of RO5126766 in a panel of human tumor cell lines [101].

A phase I open-label, dose-escalation study of RO5126766 was undertaken in 52 patients with advanced cancers [101]. Tolerability of RO5126766 was similar to that of other MEK inhibitors and the most common toxicities included rash-related disorders, elevated CPK, and diarrhea. The overall objective response rate was 40% in forty-five patients. Of 21 patients with metastatic melanoma included in the study, three PR were seen in two B-Raf mutant melanomas and one in an N-Ras mutant melanoma. The dose recommended for phase II investigation was 2.7 mg daily 4 days on/3 days off.

## WX-554

WX-554 is another MEK 1/2 inhibitor. To determine pharmacokinetic and pharmacodynamic parameters, WX-554 is planned to be administered intravenously as single doses in the range of 0.05 mg/kg to 5.0 mg/kg to healthy volunteers in dose escalated manner [102]. Results of this study are not available yet. An oral formulation of this inhibitor is being tested in a phase I/II trial in patients with advanced solid tumors (NCT01581060).

## RO4987655 (CH4987655)

RO4987655 is a highly selective, small molecule MEK inhibitor. The unique 3-oxo-oxazinane ring structure of RO4987655 confers metabolic stability, This compound showed slow dissociation from MEK with remarkable antitumor efficacy, and insignificant MEK inhibition in mouse brain, implying few CNS-related side effects in human [103].

In a recently published phase I study of RO4987655, MEK 1 inhibition in cancers was demonstrated by decreased ERK1/2 phosphorylation. Partial responses and stable disease were achieved below MTD (8.5 mg twice daily) mainly in patients with skin melanomas [104]. DLTs were reversible grade 3 blurry vision and grade 3–4 elevation of CPK. The compound alone is currently undergoing further clinical development in an expansion of this study [NCT00817518].

## GDC-0973 (XL518)

A derivative of methanone, GDC-0973 is a potent, orally bioavailable, small-molecule inhibitor of MEK 1 [105]. GDC-0973 showed strong antineoplastic activity in a B-Raf and K-Ras mutant cancer cell lines [105-107].

In a phase I clinical trial of 46 evaluable patients, GDC-0973 in combination with GDC-0941 induced PR in 3 patients (B-Raf melanoma, B-Raf pancreatic cancer, K-Ras endometrioid cancer) and stable disease in 5 [108]. Safety data showed that the DLTs were increase in serum lipase and CK enzymes. Additional phase I-III clinical trials are ongoing [NCT01689519, NCT01271803, NCT00996892, NCT01271803, NCT01562275].

## AZD8330 (ARRY-424704, ARRY-704)

AZD8330 (ARRY 704) represents a new member of MEK1/2 inhibitors [109]. A large phase I trial of 82 patients with advanced solid tumors defined the MTD to be 40 mg/day. Change in mental status was the dose limiting toxicity. Other common TEAEs include rash, fatigue, diarrhea and vomiting. Disease control rate of 40% was demonstrated in this study. Mutation analysis of Ras/Raf genes were not mandated by the study [110].

## Conclusions and future directions

Four distinct MAP kinase signaling pathways involving 7 MEK enzymes have been identified. MEK1 and MEK2 are the prototype members of MEK family proteins. Several MEK inhibitors are in clinical trials. Trametinib is being evaluated by FDA for the treatment of metastatic melanoma. Targeted therapies with small molecular inhibitors for solid tumors and hematological malignancies are moving quickly from bench to bedside [111-113]. Combination of targeting agents against different signaling pathways may provide additional benefits and warrant further clinical studies [114,115].

## Competing interests

The authors have no relevant competing interest.

## Authors’ contributions

All authors have contributed to data preparation, drafting and revising the manuscripts. All authors have read and approved the final manuscript.
